# Unveiling Lethal Risks Lurking in Hot Tub Baths: A Review of Tragic Consequences

**DOI:** 10.7759/cureus.54198

**Published:** 2024-02-14

**Authors:** Roopali Dahiya, Maryam Asif, Sharanya E Santhi, Amna Hashmi, Awranoos Ahadi, Zara Arshad, Faisal Nawaz, Rahul Kashyap

**Affiliations:** 1 Research, Global Remote Research Scholars Program, St. Paul, USA; 2 Internal Medicine, Atal Bihari Vajpayee Institute of Medical Sciences and Dr. Ram Manohar Lohia Hospital, Delhi, IND; 3 Internal Medicine, College of Medicine, Alfaisal University, Riyadh, SAU; 4 Internal Medicine, Trinity Health Oakland Hospital/Wayne State University, Pontiac, USA; 5 Internal Medicine, University of Debrecen, Debrecen, HUN; 6 Internal Medicine, Bolan Medical College, Quetta, PAK; 7 Internal Medicine, Shifa International Hospital Islamabad, Islamabad, PAK; 8 Psychiatry, Al Amal Psychiatric Hospital, Dubai, ARE; 9 Critical Care Medicine, Mayo Clinic, Rochester, USA; 10 Research, WellSpan Health, York, USA

**Keywords:** hot tub drowning, mitigation strategies, drowning, tragic deaths, hot water bath

## Abstract

Heat therapy, including saunas, jacuzzi, and hot tub bathing, has gained global popularity. However, the escalating incidents of injuries and fatalities associated with hot tub activities are a significant public health concern. This study aims to comprehensively review and analyze the pathophysiological factors contributing to hot tub-related deaths, addressing the need for awareness and mitigation strategies. A comprehensive search of electronic databases, PubMed and Science Direct, was conducted to identify articles relevant to bath-related deaths. Eligible studies were exported to the Rayyan (Qatar Computing Research Institute, Qatar) software for data analysis. The data extracted from the 18 studies were compiled to elucidate the mechanisms underlying hot tub bath-related deaths and to advocate for the adoption of potential mitigation strategies and future directions to prevent such incidents in the future. The review revealed insights into the current trend of fatalities linked to hot tub bathing. A detailed analysis of pathophysiological aspects, encompassing hemodynamics, electrolyte disturbances, serum glucagon alterations, and the impact of alcohol and substance abuse during hot tub use, was conducted. Furthermore, we explored the effects of temperature and conducted a thorough discussion of postmortem evidence analysis concerning deaths related to bathtub usage. Finally, the paper discusses mitigation strategies to prevent fatalities attributed to hot tub bathing. In conclusion, our review highlights growing public health concerns surrounding injuries and fatalities related to hot tub activities. Through an examination of the incidence rates, pathophysiological factors, and proposed mitigation strategies, we provide crucial insights for enhancing safety and addressing the escalating risks associated with hot tub bathing.

## Introduction and background

Heat therapy, such as saunas or hot tub bathing, is a popular recreational activity worldwide [1]. It is characterized by the immersion of the body in high-temperature water, typically between 41°C and 45°C, a practice akin to the Japanese custom of hot-water bathing [2]. The growing popularity of hot tub bathing over the past two decades has also led to an alarming rise in bathing-related injuries and fatalities [3]. In Japan alone, approximately 14,000 bath-related deaths are reported annually, accounting for over 15% of sudden out-of-hospital fatalities [4]. Similarly, in the US, there has been a significant increase in the number of unattended adult deaths due to bathtubs. Notably, from 1990 to 2007, over 80,000 individuals suffered severe injuries in hot tubs or whirlpools, with nearly 74% of these incidents occurring at home [3].

The prevalence of sudden deaths in hot tubs has been linked to common factors such as drowning in combination with narcotic intoxication, cardiovascular issues, drowning with a concomitant attack of unconsciousness, and even natural causes unrelated to drowning. Among these, accidental bathtub drowning is one of the leading causes of death [5]. Consumption of alcohol during sauna bathing increases the risk of low blood pressure, fainting, arrhythmia, and sudden death, particularly in individuals with underlying coronary heart disease [6]. Almost all (221 of 228) hyperthermia deaths in Finland from 1970 to 1986 occurred in saunas and were considered accidents. Most victims were middle-aged men, 84% were under the influence of alcohol, and 27% had cardiovascular diseases (CVDs) [6]. Elevated heat in hot baths can lead to cardiovascular complications, such as ventricular tachycardia, ventricular extrasystole, or a drop in blood pressure, consequently triggering fatal cardiac events or drowning with an attack of unconsciousness [5].

Recent research has highlighted the potential benefits of heat exposure in tub bathing as a preventative measure against CVD [4,6,7]. It has been implicated in enhancing lung function among individuals with obstructive airway disease and alleviating pain in patients with rheumatoid arthritis [6]. However, recent high-profile incidents, such as the unintentional drowning of Bollywood actress Sridevi in a hot tub in Dubai, United Arab Emirates, in 2018 [8], and the premature death of Matthew Perry, a star of the popular American television sitcom "Friends," in Los Angeles, USA, in October 2023 due to accidental drowning, have highlighted the necessity of understanding the pathophysiological aspects of such occurrences and addressing them as a pressing public health issue [9].

## Review

Materials and methods

**Figure 1:**
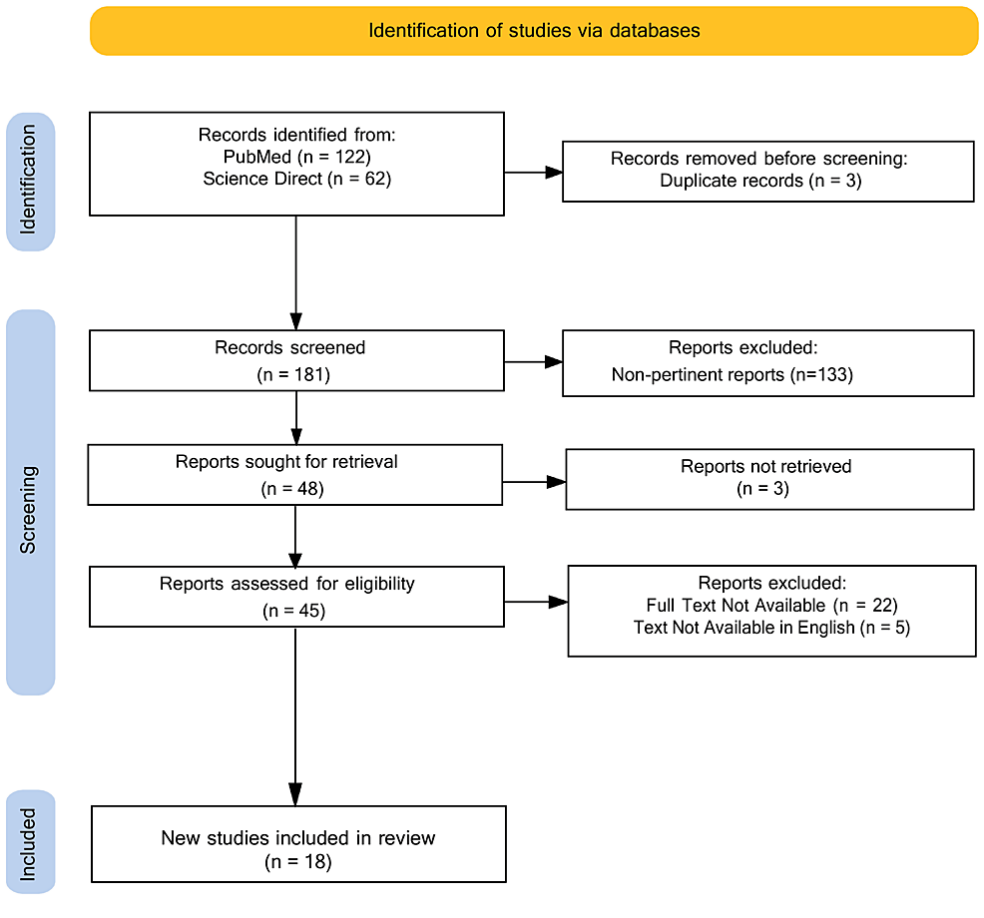
Flow diagram Flow diagram of article selection

We used the electronic scientific resources PubMed and Science Direct to gather data on bathtub-related deaths. The search involved relevant English articles employing keywords such as "bath related deaths," "hot tub," "bathing," "sauna," "hot spring," and "heat therapy," along with terms such as "drowning" or "accidental drowning." Additionally, the search included terms such as "spa-related injuries," "bathtub drowning," and "sudden death" to ensure comprehensive coverage of relevant studies. The search was performed by five authors (RD, MA, SS, AH, and AA) using the Rayyan (Qatar Computing Research Institute, Qatar) software. The correctness of the document titles was verified, and duplicates were eliminated. In total, 184 articles were discovered using our search strategy, and three articles were omitted due to duplication. Following the screening of article titles and the abstracts, 133 non-pertinent articles were excluded, leaving us with a total of 48 articles for full-text screening. Among these, 22 articles were not available in full text, and five were not in English. Finally, 18 articles were included in this review (Figure 1). Any conflicts between the authors were resolved through discussion, and, if necessary, an additional third arbiter was consulted.

Incidence

Addressing the current trend of bathtub drowning among adults in the US is a matter of concern and vital for the safety and well-being of individuals across all age groups. The increasing indulgence of our young population in drug abuse [10] and the growing aging population in the US [11] make bathing, especially in a bathtub, more challenging and increases the risk of accidents, including drowning. Being vigilant about the risks associated with basic activities of daily living, such as bathing, among adults can help prevent tragic deaths in the future.

The recent death of Matthew Perry underscores the need for safety precautions and awareness, even in seemingly routine situations. These lessons can serve as a reminder to value and cherish life while working to prevent accidents, health issues, and substance abuse and to seek help and support when facing mental health challenges.

In the existing literature, we encountered numerous studies investigating the factors and findings associated with bathtub-related fatalities in Japan compared with other nations [12]. This disparity is likely attributed to Japan’s distinct bathing practices, where mostly elderly individuals immerse themselves up to the shoulder level in deep bathtubs filled with hot water at 42°C or higher during the winter months [12].

As per the retrospective study by Xu J [13] on unintentional deaths due to drowning in the US from 1999 to 2010, natural water was the most frequent drowning location, accounting for 47.2% of all unintentional drowning deaths (including drowning deaths while boating), followed by other/unspecified places (26.8%), swimming pools (16.3%), and bathtubs (9.7%). The average number of deaths from drowning on weekends was nearly 48% higher than that on weekdays.

Pathophysiology

Hemodynamics

Several mechanisms have been implicated in the occurrence of hot tub-related deaths. Immersion in heated water causes a drop in systolic and diastolic blood pressure with a concomitant rise in heart rate [14]. Although this is a temporary effect that resolves a few minutes after one leaves the hot tub, prolonged exposure could lead to pre-syncopal and syncopal episodes. Shin TW et al.’s study [14] demonstrated its effects on blood pressure. They noted that no symptoms, such as dizziness, palpitation, or chest pain, were reported in either stable hypertensive (study group) or normotensive patients (control group) during and after immersion in the hot tub for 10 minutes. Increased body temperature also leads to an increased metabolic demand in the brain [15]. The silent nature of pathophysiological changes could be a key factor in preventing people from removing themselves from the bathtub earlier. Syncopal episodes are generally benign and recover quickly owing to the return of optimal cerebral blood flow with postural changes. However, this could be deadly in a water body since the syncopal episode leads to aspiration, laryngospasm, and drowning when unnoticed or unmonitored [16]. Cardiac arrest, myocardial infarction, stroke, and other acute events can occur at any age or time. Igarashi T conducted a study that observed electrocardiographic changes in individuals immersed in hot water, as well as upon standing up, despite their lack of symptoms [17]. These events could result in loss of consciousness and may lead to drowning if immediate assistance is not provided. Other causes of death that occur in more elaborate settings, such as jacuzzi or pools, include electrical injuries and suction entrapment [18].

Between 2006 and 2019, Kagoshima Prefecture [2] reported 2,689 bath-related deaths. It was evident that the mortality rate of bath-related deaths increased with age. This is because only 9% of the individuals were considered healthy, and the remaining majority of patients had some past illness, including hypertension, CVD, diabetes mellitus, and a central nervous system disorder. Hypertension was the most common pre-existing condition.

Electrolytes

Changes in serum electrolyte levels are minimal and not considered clinically significant despite sauna-induced dehydration. A study in Finland measured the effects of sauna bathing on serum potassium and calcium levels. Serum potassium increased slightly the following day, whereas serum calcium increased linearly [19]. In a notable case report from Ueno D et al. [20], a rather astonishing incident unfolded when a 55-year-old woman was discovered floating in a Japanese hot spring bath. Subsequent evaluation revealed severe hypercalcemia resulting from the inadvertent ingestion of calcium-rich hot spring water. This case emphasizes the importance of recognizing the unanticipated medical consequences associated with leisure activities.

Glucagon

A Slovakian study [21] found that sauna treatment increased plasma glucagon levels, likely due to adrenergic stimulation during hyperthermia, supported by elevated catecholamine and glucagon concentrations. Factors such as reduced hepatic and renal glucagon degradation, along with diminished visceral blood flow, are likely to contribute to higher plasma glucagon levels. Presumably, due to sympatho-adrenal activity and glucagon-stimulated glycogenolysis, there was a slight rise in plasma glucose but no hypoglycemia. However, this increase in glucose levels led to a decline in plasma glucagon and adrenaline levels after the 20th minute of the 30-minute sauna session.

Alcohol and Substance Abuse

In the context of evaluating the effects of alcohol intoxication and substance abuse in conjunction with hot water bathing, a previous study [19] revealed alarming insights. Exposure to heat stress led to a 62-93% increase in the mean heart rate, accompanied by a body temperature rise of 1.5-1.8°C. Interestingly, the combination of sauna exposure and alcohol consumption resulted in a slightly greater increase in heart rate, consistent with the known impact of alcohol [19]. Another notable Finnish study reported that alcohol intoxication was the primary cause of death in 8.7% of the cases, primarily affecting males (74%). These patients exhibited an average blood alcohol content (BAC) of 0.306 g/dL, with a range of 0.169-0.401 g/dL. Furthermore, the steam room had the highest number of deaths owing to alcohol poisoning, many of which were linked to heat exposure. The study also noted instances of carbon monoxide poisoning, drug-induced suicides, and unintentional heroin overdoses. The investigation showed an increasing presence of alcohol in sauna-related deaths, with 50% of recent cases having alcohol in their blood, compared to 30% in the past. Fatal alcohol intoxication can result from central nervous system depression or the impact of alcohol on cardiac function, particularly when combined with prolonged heat exposure. Although alcohol consumption plays a significant role in sauna deaths, fatal falls and burns are rare. Recent studies have shown low prevalence and mortality rates of burns; however, sauna heat alone can cause severe tissue damage, often involving alcohol, resulting in a high mortality rate [22].

Impact of Temperature

The correlation between bathwater and ambient temperatures significantly influences bath-related fatalities. Multiple studies have established a robust association between lower air temperatures and bath-related cardiac arrests, prompting proactive preventive measures during colder forecasts [23,24]. Conversely, extreme bathwater temperatures disrupt thermoregulation, underscoring the risks of prolonged exposure to hot water [25]. Additionally, Eshel GM et al.'s animal study brought attention to the possibility of severe secondary complications, such as hemorrhagic diathesis and irreversible shock, occurring after apparent stabilization following exposure to extreme hot bath temperatures. [26].

In addition to the bath temperature, high relative humidity is a key contributor to extensive burn injuries in sauna users. Steam generation in saunas diminishes oxygen levels, potentially inducing loss of consciousness and worsening burn injuries. These findings underscore the crucial role of temperature control and humidity management in averting heat-related incidents [27].

Postmortem Evidence Analysis

Elderly victims in their late 80s and 90s often bypass autopsies due to their pre-existing medical histories providing plausible explanations for sudden death such as ischemic heart disease. Additionally, interpreting water inhalation signs during autopsy presents challenges as they may be absent even in drowning cases, owing to laryngeal spasm triggered by minimal water, mediated by the vagal reflex. This discrepancy may lead to an underestimation of drowning instances.

Suzuki H et al. conducted an evidence-based analysis investigating bathtub-related fatalities. Of the examined cases, circulatory system diseases accounted for over 54.5% of the fatalities. The prevalent pathological findings included coronary artery stenosis and cardiomegaly, identified in 43.5% of the cases, followed by atherosclerotic lesions in the cerebral arteries (5.3%). Intriguingly, approximately one-third of patients displayed no distinctive pathological findings. Notably, cases exhibiting both water inhalation signs and blood ethanol levels >0.5 mg/mL indicated the potential role of alcohol intake in cases without notable autopsy findings except for drowning [28].

Additionally, Oshima T's study drew attention to the manifestation of subcutaneous back hemorrhages without accompanying muscular hemorrhages around the scapula - an identifiable autopsy sign seen in bathtub-related deaths. The study highlighted orthostatic hypotension upon exiting the bathtub as a potential instigator of this distinctive marker [29]. These studies emphasize the critical need for additional specific diagnostic indicators to accurately determine the cause of death in bathtub-related deaths.

Mitigation

Several strategies have been proposed to mitigate bathtub-related deaths, including improving safety measures, raising awareness about potential drug and medication side effects, screening individuals for underlying health conditions, conducting regular assessments of patients with pre-existing conditions, providing guidance on safe bathing practices, and installing bath aids to ensure safety in emergency situations. Safety precautions include installing non-slip mats and bathtub safety handles, avoiding extremes of water temperature for bathing, using bath seats for those with disabilities or limitations, and installing emergency alert systems in case assistance is needed (Table 1). Additionally, it is vital to provide comprehensive guidelines for caution against alcohol consumption in hot tubs and enforce strict policies prohibiting recreational drug use in these areas [30,31]. It is recommended that individuals with pre-existing comorbidities, such as coronary artery disease, cardiac arrhythmias, and autonomic dysfunctions (e.g., diabetes, Parkinson’s disease, multiple sclerosis, and amyloidosis), or pregnant women, consult with healthcare providers before using hot tubs. Encouraging awareness of warning signs, necessitating immediate cessation of hot tub use, ensuring adequate hydration, and prompting users to disclose any pre-existing conditions to companions or staff for timely assistance are key. Moreover, training staff to recognize distress or mental health concerns and promote mental health awareness to discourage hot tub use during heightened emotional states significantly contributes to overall safety and well-being in hot tub facilities.

**Table 1:** Mitigation strategies for hot tub bath-related emergencies

Mitigation strategies	Details
Safety precautions	Installing non-slip mats and bathtub safety handles.
	Temperature control: avoiding extremes of water temperature for bathing.
	Encourage users to limit their time in the hot tub to avoid adverse outcomes.
	Use of bath seats for those with disability or limitations.
	Supervision: always supervise children and vulnerable individuals in or around a hot tub.
	Avoid playing loud music to facilitate assistance in times of need.
	Installing emergency alert systems.
Alcohol consumption	Provide clear guidelines on the risks of combining alcohol with hot tub use.
	Ensure rules that limit or prohibit alcohol consumption in or around hot tubs.
Recreational drugs	Enforce a strict zero-tolerance policy for drug use in and around hot tubs.
	Implement random checks to discourage drug use and identify potential risks.
Pre-existing conditions	Enforce policies requiring individuals with pre-existing comorbidities to consult their healthcare provider before hot tub use.
	Encourage patients to be aware of any warning signs or symptoms that warrant immediate cessation of hot tub use.
	Maintain adequate hydration before, during, and after hot tub use.
	Encourage users to disclose any pre-existing conditions to companions or hot tub staff for timely assistance.
Mental health care	Train staff to recognize signs of distress or mental health concerns and provide appropriate assistance.
	Display signs promoting mental health awareness and encouraging individuals to refrain from hot tub use during heightened emotional states.

Future Direction

Next-generation DNA sequencing offers the potential to identify individuals at risk of arrhythmogenic fatalities in hot tubs, possibly influenced by multiple channelopathy-related gene variants and alcohol consumption, as reported by Hata Y et al. [32]. Future DNA sequencing analysis may help to predict such risks. Nonetheless, implementation of widespread genetic testing solely for hot tub safety remains an evolving area of study.

**Figure 2:**
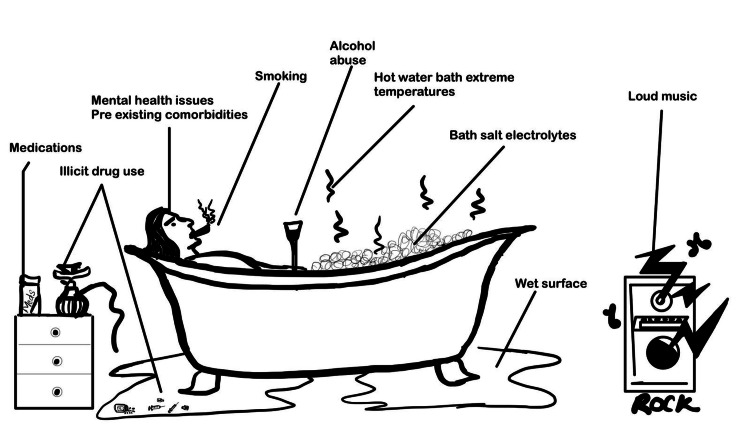
Potential causes contributing to hot tub-related deaths This figure is an authors' creation.

Several mechanisms have been implicated in the occurrence of hot tub-related deaths (Figure 2).

## Conclusions

Recognizing the importance of this issue, the introduction of public health education can help reduce the incidence of accidental deaths caused by bathtub drowning, thereby promoting safety both within households and outdoor settings. Analyzing the factors that contribute to bathtub drowning can yield valuable insights into the medical aspects of these cases. This includes understanding the role of comorbidities, drug abuse, medication-related adverse effects, mental health care, and genetic screening in individuals with a risk of arrhythmias during and after hot tub baths.
